# *Aquilaria sinensis*: An Upstart Resource for Cucurbitacin Production Offers Insights into the Origin of Plant Bitter (*Bi*) Gene Clusters

**DOI:** 10.3390/plants13020260

**Published:** 2024-01-16

**Authors:** Xupo Ding, Zhuo Yang, Hao Wang, Jun Zeng, Haofu Dai, Wenli Mei

**Affiliations:** 1 Key Laboratory of Research and Development of Natural Product from *Li Folk* Medicine of Hainan Province, Institute of Tropical Bioscience and Biotechnology, Chinese Academy of Tropical Agricultural Sciences, Haikou 571101, Chinawanghao@itbb.org.cn (H.W.);; 2 International Joint Research Center of Agarwood, Institute of Tropical Bioscience and Biotechnology, Chinese Academy of Tropical Agricultural Sciences, Haikou 571101, China; 3 Hainan Engineering Research Center of Agarwood, Institute of Tropical Bioscience and Biotechnology, Chinese Academy of Tropical Agricultural Sciences, Haikou 571101, China

**Keywords:** cucurbitacin, *Aquilaria sinensis*, *Begonia*, *Bi* gene, bitter gene cluster, origin, evolution

## Abstract

Cucurbitacins, oxygenated tetracyclic triterpenoids that are found mainly in the Cucurbitaceae family, play essential roles as defensive compounds, serving as allomones against herbivores and pathogens and as signals for insect–parasite recognition. These compounds also exhibit various pharmacological effects. The biosynthesis of cucurbitacins is largely regulated by the bitter (*Bi*) gene, encoding an oxidosqualene cyclase, which catalyzes the conversion of 2,3-oxidosqualene into cucurbitadienol, a common precursor for cucurbitacin synthesis. Previous studies focused on uncovering the *Bi* gene clusters in Cucurbitaceae, but their presence in other cucurbitacin-producing plants remained unexplored. Here, the evolutionary history of *Bi* genes and their clusters were investigated in twenty-one plant genomes spanning three families based on chemotaxonomy. Nineteen *Bi* genes were identified in fourteen Cucurbitaceae, four Begoniaceae, and one *Aquilaria* species. Phylogenetic analysis suggested that the genome of *Aquilaria sinensis* contained the earliest *Bi* gene clusters in this dataset. Moreover, the genomic analysis revealed a conserved microsynteny of pivotal genes for cucurbitacin biosynthesis in Cucurbitaceae, while interspersed *Bi* gene clusters were observed in Begoniaceae, indicating rearrangements during plant *Bi* gene cluster formation. The bitter gene in *A. sinensis* was found to promote cucurbitadienol biosynthesis in the leaves of *Nicotiana benthamiana*. This comprehensive exploration of plant *Bi* genes and their clusters provides valuable insights into the genetic and evolutionary underpinnings of cucurbitacin biosynthesis. These findings offer prospects for a deeper understanding of cucurbitacin production and potential genetic resources for their enhancement in various plants.

## 1. Introduction

Cucurbitacins (Cu) are characteristic oxygenated tetracyclic triterpenoids that are mainly present in the Cucurbitaceae family and are arbitrarily divided into 12 categories (cucurbitacins A–T), among which Cu B is ubiquitously distributed in this family of plants [1]. Being allomone in nature, cucurbitacins have a protective function in plants against the attacks of herbivores and pathogens. For example, the content of Cu C in cucumber was significantly correlated with resistance against *Tetranychus urticae* [2]. Cucurbitacins are also exploited as signals by insects to recognize parasites. Their contents can increase quickly once the plants are foraged by herbivores to induce an antifeedant effect [3]. Previous studies have also indicated that Cu B acts as a potential growth regulator for insects by antagonizing 20-hydroxyecdysone activity [4]. Cucurbitacins also show pharmacological effects, as they could have cytotoxic, purgative, anti-inflammatory, and anti-fertility activity [1,5].

**Figure 1:**
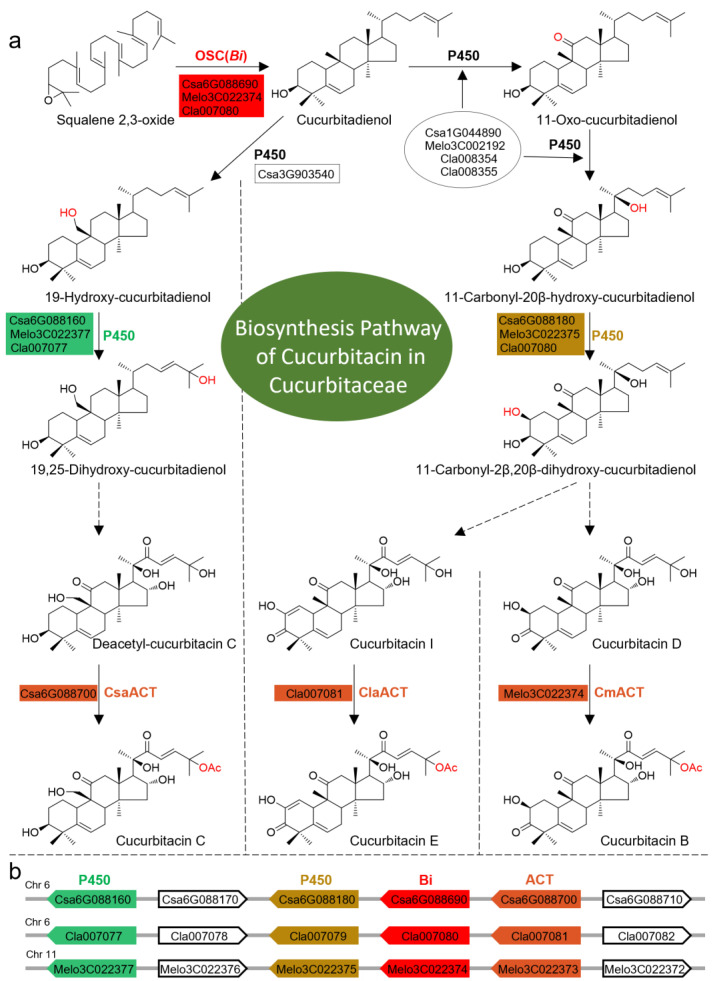
The biosynthesis pathway of cucurbitacins in Cucurbitaceae and an illustration of the *Bi* gene cluster in the cucumber genome. (**a**) The biosynthesis pathway of cucurbitacins in Cucurbitaceae. The enzyme names, colored with red, green, brown, and mustard, are distributed in the *Bi* gene cluster and the gene IDs to the left of the enzymes are those corresponding to the IDs in the genomes of cucumber, watermelon, or melon. OSC: oxidosqualene cyclase; *Bi*: bitter gene; P450: cytochrome P450; ACT: acyltransferase; Csa: *Cucumis sativus*; Melo/Cm: *Cucumis melo*; Cla: *Citrullus lanatus*; Ac: acetyl. (**b**) A schematic diagram of *Bi* gene clusters and gene orders distributed on chr 6 in the cucumber genome, chr 6 in the watermelon genome, and chr 11 in the melon genome. The genes with the same color background are the syntenic genes in these three species from the *Cucumis* genus. The gray line in the background represents the chromosome and the arrow represents the transcriptomic direction of each gene. Chr: chromosome.

The bitter taste of *Cucumis sativus* is from cucurbitacins and their biosynthesis is majorly rate-limited by the bitter (*Bi*) gene, which encodes a type of oxidosqualene cyclase (OSC). OSC catalyzes 2,3-oxidosqualene to generate cucurbitadienol, which acts as a common precursor for the biosynthesis of various cucurbitacins in plants (Figure 1a) [6]. The whole genome sequence of cucumber revealed a *Bi* gene cluster involved in cucurbitacin biosynthesis [6]. Further, nine genes involved in the biosynthesis of cucurbitacin C were identified in cucumber (Figure 1a) [7]. Subsequently, *Bi* gene clusters were also found in melon and watermelon. These functional genes, especially OSC (*Bi*), P450 encoding C25 or 2β hydroxylase, and acyltransferase (ACT), in *Bi* gene clusters are distributed in a collinear manner in different species of Cucurbitaceae, even showing the same order in their arrangements and direction of transcription across various species (Figure 1b) [8]. These observations suggest that *Bi* gene clusters might be highly conserved during the process of plant evolution. This hypothesis could not be investigated fully as the previous studies were focused on the species from Cucurbitaceae, whereas the *Bi* genes and their clusters in other species that produce cucurbitacins have not been investigated.

Plant chemotaxonomy provided the key information about the distribution of cucurbitacins, which can also be detected or extracted from the species belonging to Begoniaceae, Brassicaceae, Celastraceae, Elaeocarpaceae, Euphorbiaceae, Polemoniaceae, Rubiaceae, Scrophulariaceae, Sterculiaceae, Tetramelaceae, or Thymelaeaceae [1]. This implies that such species might also contain a *Bi* gene or *Bi* gene clusters in their genomes. Our previous study first reported that Cu D and Cu I were isolated from the fruits of *Aquilaria sinensis* [9]. Also, Cu E and Cu I were detected from callus, shoot, and in vitro plants of *A. agallocha*, and even the content of Cu E reached 1.235 mg/g in leaves after treatment with 0.5 mM methyl jasmonate [10]. These studies suggested that *Aquilaria* species might be an upstart natural resource for cucurbitacin production. In the last 10 years, an increasing number of plant genomes, including that of *A. sinensis*, have been sequenced [11]. They provide a useful resource for exploring the distribution of *Bi* genes and their clusters in the plant kingdom.

In this study, the *Bi* gene was first identified in the *A. sinenesis* genome with CLEAN [12], an annotation script of a machine learning algorithm. Then, plantiSMASH was employed to analyze a short scaffold containing this *Bi* gene belonging to the *A. sinensis* genome, resulting in this scaffold including a typical terpene gene cluster, which presents a characterization of the classical *Bi* gene clusters from the species of Cucurbitaceae. Subsequently, 20 other genomes from 2 plant families, of which 16 were from Cucurbitaceae and 4 were from Begoniaceae, based on the chemotaxonomy that they all produce cucurbitacin, were selected to present the evolution of plant *Bi* genes and *Bi* clusters together with the *A. sinensis* genome. The evolutionary history of these species suggested that plant *Bi* gene clusters may have originated from *A. sinensis* in this dataset. Finally, the *Bi* genes from the *A. sinensis* genome were demonstrated to promote cucurbitadienol biosynthesis. Understanding the evolutionary and genetic basis of plant *Bi* genes and their clusters could contribute to the biosynthetic pathway resolution of multifarious cucurbitacins thoroughly and provide crucial genetic or germplasm resources for cucurbitacin production.

## 2. Results and Discussion

### 2.1. Characterization of Plant Bitter Genes

To identify the bitter genes, a machine learning algorithm of CLEAN [12] was employed to screen the protein sequences to identify the *Bi* genes in 21 plant genome datasets. These species were selected from three families on the basis of their chemotaxonomy (cucurbitacin production). The results of CLEAN were verified by Blastp [13] and HmmScan [14]. Finally, a total of 19 *Bi* genes, annotated to code for a cucurbitadienol synthase (EC: 5.4.99.33; *p*-value > 0.95), were retained from 14 species of Cucurbitaceae, 4 from Begoniaceae, and 1 from *Aquilaria*. Generally, these genes in the Pfam database [15] synchronously contained both the domains of PF13249.9 (SQHop_cyclase_N) and PF13243.9 (SQHop_cyclase_C). In the *A. sinensis* genome dataset, *As03G2784* was identified and annotated as EC: 5.4.99.33 (0.97) with CLEAN, and it simultaneously contained two conserved domains from Pfam. No bitter genes were identified in *Benincasa hispida* or *Lagenaria siceraria*.

**Figure 2:**
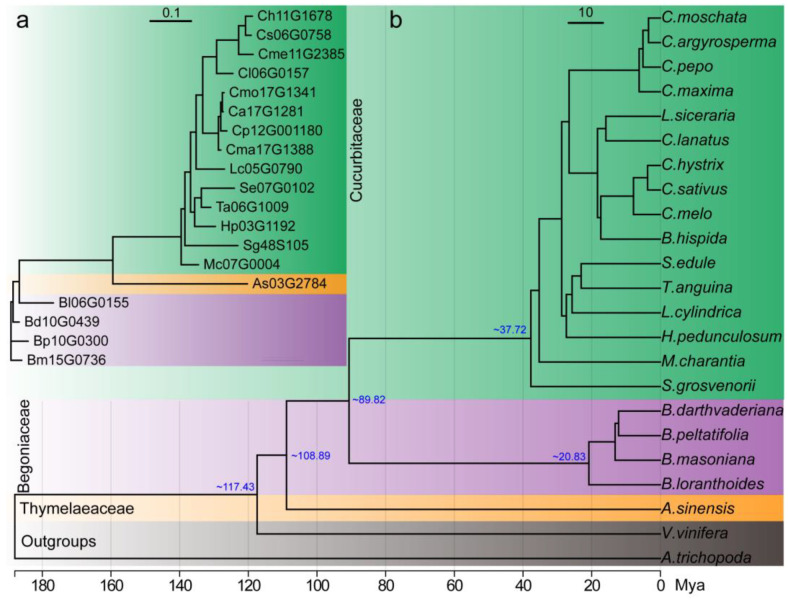
Phylogenetic analysis of the plant bitter genes and their species. (**a**) Phylogenetic analysis of the plant bitter genes. The evolutionary trees were constructed by IQ-tree2 with the best model of JTT+I+I+R2. (**b**) Single copy gene tree of 21 plant species that produce cucurbitacin and two model species of *Amborella trichopoda* and *Vitis vinifera* as the outgroups. The blue numbers around the nodes are the approximate divergence times of the species. Mya: million years ago.

To characterize the evolutionary relationships of the plant *Bi* genes, a maximum likelihood tree was created with IQ-tree2 by the best model of JTT+I+I+R2. Nineteen *Bi* genes were classified into three subfamilies, which was consistent with the plant taxonomy; the *Bi* genes from the Begoniaceae family were located in the root section of the phylogenetic tree (Figure 2a). But in a macroevolutionary tree of the 23 species, the origin time of Cucurbitaceae and Begoniaceae was around 37.72 million years ago (Mya) and 20.83 Mya, respectively. These are later than the origin time of *A. sinensis*, which is around 108.89 Mya (Figure 2b). These divergence times of the species are similar to those in previous reports [11,16,17]. As the outgroups, *Amborella trichopoda* and *Vitis vinifera* did not contain the *Bi* gene in their genomes.

### 2.2. Identification and Microsynteny of Bitter Gene Clusters

To investigate the occurrence of a *Bi* gene cluster in the *A. sinensis* genome, the genes distributed on a single scaffold containing *As03G2784* (Scaffod63), before the Hi-C anchoring, were re-analyzed. The total length of Scaffod63 was 2.15 Mb and it included 22 tactic genes (*As03G2769*-*As03G2790*), of which 15 had a homolog in a public database. Nine other genes besides *As03G2784* might be associated with secondary metabolite biosynthesis or transport. *As03G2774*, *As03G2778*, *As03G2780*, *As03G2786*, and *As03G2788* were classified as cytochrome P450s (P450), *As03G2777* was identified as BAHD acyltransferase (ACT), and *As03G2781* was annotated as an oxidoreductase (OR). *As03G2770* and *As03G2772* were identified as an ABC and a MATE transporter, respectively.

**Figure 3:**
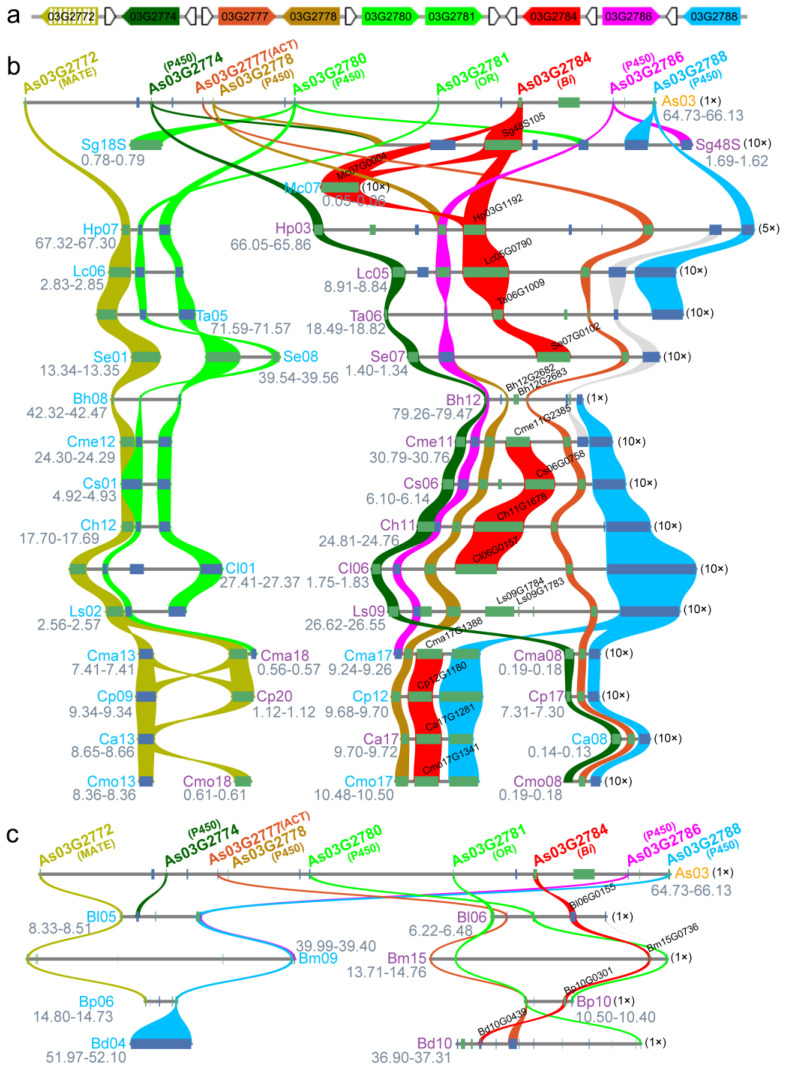
A bitter gene (*Bi*) cluster on chr 3 of the *A. sinensis* genome and its microsynteny with clusters of species from Cucurbitaceae and Begoniaceae. (**a**) Schematic diagram of bitter gene (*Bi*) clusters on chr 3 of the *A. sinensis* genome. The gene named as As03G2772, in hatched grass yellow, is not in the typical gene cluster from the predication by plantiSMASH; the genes uncolored were annotated as uncharacterized proteins. (**b**) Microsynteny of bitter gene clusters between *A. sinensis* and the species from Cucurbitaceae. MATE: multidrug and toxic compound extrusion transporter; P450: cytochrome P450; ACT: acyltransferase; OR: oxidoreductase; *Bi*: bitter gene. Sg: *Siraitia grosvenorii*; Mc: *Momordica charantia*; Hp: *Herpetospermum pedunculosum*; Lc: *Luffa cylindrica*; Ta: *Trichosanthes anguina*; Se: *Sechium edule*; Bh: *Benincasa hispida*; Cme: *Cucumis melo*; Cs: *Cucumis sativus*; Ch: *Cucumis hystrix*; Cl: *Citrullus lanatus*; Ls: *Lagenaria siceraria*; Cma: *Cucurbita maxima*; Cp: *Cucurbita pepo*; Ca: *Cucurbita argyrosperma*; Cmo: *Cucurbita moschata*. (**c**) Microsynteny of bitter gene clusters between *A. sinensis* and the species from Begoniaceae. Bl: *Begonia loranthoides*; Bm: *B. masoniana*; Bp: *B. peltatifolia*; Bd: *B. darthvaderiana*. The number in the parentheses is the magnification for gene cluster visualization.

Then, Scaffold63 and its gff3 files were screened by plantiSMASH 1.0 and the results indicated that 1.38 Mb of Scaffold63 was a typical plant terpene biosynthesis cluster that contained 16 genes, from *As03G2773* to *As03G2788* (Figure 3a). The microsynteny analysis demonstrated that the six pivotal genes for cucurbitacin biosynthesis in Cucurbitaceae species showed collinear relationships with the *Bi* gene cluster from *A. sinensis*, including a *Bi* gene, four P450s, and an ACT (Figure 3b). In *Momordica charantia*, only the *Bi* gene presented collinearity with other species, whereas the conserved synteny gene pairs as P450s or ACT homologs were not similarly detected in this species [18]. A potential *Bi* gene in *B. hispida* was interrupted by an insertion of 864 bp non-coding sequences between *Bh12G2682* (SQHop_cyclase_N) and *Bh12G2683* (SQHop_cyclase_C), which would have formed two domains of the *Bi* protein. Now, this insertion has split the potential *Bi* gene in *B. hispida* into two separate genes that might have resulted in the *Bi* gene being lost in the wax gourd. The same characteristics were also found in the *L. siceraria* genome, which had a 3043 bp insertion of non-coding sequences between *Ls09G1383* (SQHop_cyclase_N) and *Ls09G1784* (SQHop_cyclase_C) (Figure 3b). The core biosynthesis clusters of cucurbitacins in the *Cucurbita* genus were separated into two chromosomes and lost the homolog of *Cs06G0755* (*Csa6G0088170*) except in *C. maxima*, but there were two homologous genes of *Cs06G0760* (Csa6G0088710) in the *Cucurbita* genus (Figure 3b). A syntenic pair of a MATE gene (*Csa1G044870*/*Cs01G0775*) contributing to cucurbitacin transport was also found in the nearby region of the *Bi* gene cluster from *A. sinensis* [19], although *As03G2772* was not previously identified as a member of a typical plant biosynthesis gene cluster (Figure 3b). 

We inferred that a total of eight syntenic genes are involved in cucurbitacin biosynthesis and transport, which are present on Scaffold63 of the *A. sinensis* genome, except for *As03G2772* (Figure 3a). It is interesting that a P450 gene (*Csa1G044890*/*Cs01G0777*), involved in cucurbitacin C biosynthesis [7], was found around this MATE gene in the cucumber genome. But the collinear pair of *Csa1G044890*/*Cs01G0777*, a syntenic gene of *As03G2780*, could not be detected around a MATE transporter in the four species of *Cucurbita* (Figure 3b).

Unlike the compact *Bi* gene clusters in Cucurbitaceae, the *Bi* gene clusters in Begoniaceae were interspersed, especially for the collinear gene pairs of six key biosynthesis genes for cucurbitacin C biosynthesis from cucumber. The syntenic pairs of two key P450s named *Cs06G0759* (*Csa6G0088700*) and *Cs06G0760* (*Csa6G088710*) were not distributed around the *Bi* genes in *Begonia* species, but were near a MATE transporter gene from another chromosome (Figure 3c). All four *Begonia* species do not contain a synteny pair of *Cs06G0754* (*Csa6G088160*). In addition, the collinear pairs of P450 named *Cs06G0759* (*Csa6G0088700*) were also lost in *B. peltatifolia* and *B. darthvaderiana* (Figure 3c). These results suggested that rearrangements by crossover or insertion between different chromosomes and gene loss have occurred during plant *Bi* gene cluster formation in different species.

### 2.3. Bitter Gene in A. sinensis Promotes Cucurbitadienol Biosynthesis in the Leaves of N. benthamiana

**Figure 4:**
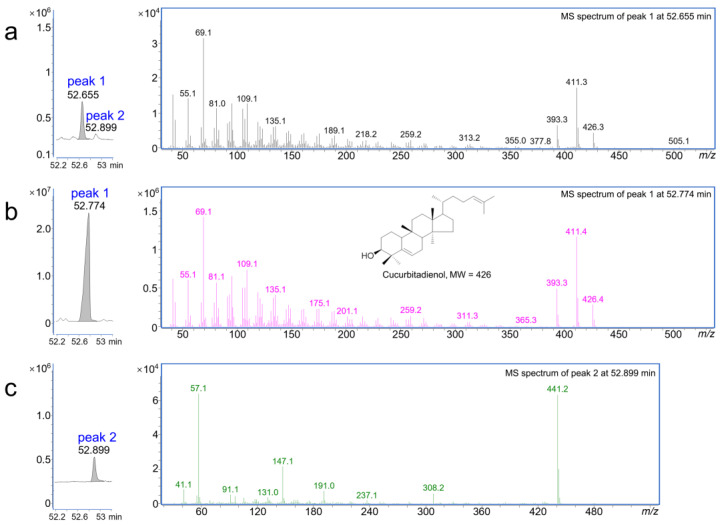
GC-MS determined the function of *As03G2784* by transient expression in the leaves of *Nicotiana benthamiana*. (**a**) MS chromatograms (EIC) of *N. benthamiana* transiently expressing *As03G2784* (*Bi*) (**left**) and the EI–MS spectrum of peak 1 (**right**). (**b**) MS chromatogram (EIC) of the cucurbitadienol standard (**left**) and its EI–MS spectrum (**right**). (**c**) MS chromatogram of *N. benthamiana* transiently transfected with empty vector only (**left**) and the EI–MS spectrum of peak 2 (**right**).

To explore the function of the *Bi* gene in *A. sinensis*, the full-length ORF of *As03G2784* was cloned and inserted into the *p*Cambia1300-35S vector to generate the overexpression plasmid of *p*Cambia1300-35S-*As03G2784*. Then, a strain of *Agrobacterium tumefaciens* containing *p*Cambia1300-35S-*As03G2784* was infiltrated into the leaves of *Nicotiana benthamiana* for transient expression. One new peak at 52.655 min was detected in re-suspension liquid from an extract of the transfected *N. benthamiana* leaves (Figure 4a) by using the gas chromatography–mass spectrometry (GC-MS) system of Agilent 7820A-5977E compared with another peak named peak 2 at 52.899 min in all treatments except the cucurbitadienol standard (Figure 4 and Figure S1). The retention time of this new peak was nearer the retention time of 52.774 min of the cucurbitadienol standard (Figure 4b). Furthermore, the MS spectrum of peak 1 at 52.655 min from the transfection treatment (Figure 4a) was similar to the MS spectrum of peak 1 at 52.774 min of the cucurbitadienol standard (Figure 4b), and both presented the same electron ionization (EI) spectrum (*m*/*z* 426 [M]^+^, 411 [M−CH_3_]^+^, 393 [M−CH_3_−H_2_O]^+^, 109 [C_8_H_13_]^+^, 69 [C_5_H_9_]^+^, etc.) (Figure 4a,b). Peak 2 at 52.899 min in all treatments except the cucurbitadienol standard also presented the same electron ionization (Figure 4 and Figures S1–S5). In contrast, the characteristic fragment ions of the cucurbitadienol standard were not found in the spectra of two control treatments or in peak 2 (Figure 4c and Figures S1–S5). This result indicated that peak 1 at 52.655 min in Figure 1a might represent the compound cucurbitadienol and suggested that the *Bi* gene in *A. sinensis* could also promote cucurbitadienol biosynthesis.

## 3. Materials and Methods

### 3.1. Datasets and Plant Materials

The genome files of *Amborella trichopoda* and *Vitis vinifera* were collected from JGI [20]. The genome files of four *Begonia* species (*B. loranthoides*, *B. masoniana*, *B. peltarifolia*, and *B. peltarifolia*) were downloaded from CNGdb (https://db.cngb.org/, accessed on 21 August 2023), and the genome files of 16 gourd species were selected from CuGenDB [21], whereas the genome file of *A. sinensis* was from our previous study [11]. All genes were renamed by their order in pseudo chromosomes or scaffolds based on the bed format.

The functional gene transformation was performed on tobacco (*Nicotiana benthamiana*) plants that were grown in a growth chamber for 40 days. The plants were grown in pots filled with a soil and vermiculite mixture (2:1) under a photoperiod cycle of 16 h lightness and 8 h darkness at 25 °C and 70% relative humidity [17]. The seeds of *N. benthamiana* were obtained from the laboratory of Dr. Yuan Yao in our institute.

### 3.2. Identification and Evolution of Bitter Genes and Gene Clusters

A machine learning model, CLEAN (Contrastive Learning-enabled Enzyme Annotation) [12], was used to capture molecular features of proteins from amino acid sequences with the default parameters from 23 species at the genome-wide level. The target bitter genes were simultaneously annotated to two enzyme commission (EC) categories of EC: 5.4.99.8 and EC: 5.4.99.33, which were respectively classified as cycloartenol synthase and cucurbitadienol synthase in the EC number database. These sequences are presented in Table S1. Then, IQ-tree2 was employed to construct a phylogenetic tree of all bitter genes identified above with the following parameters: -m MFP -B 1000 --bnni -T AUTO [22]. The collinearity pairs of *Bi* and its nearby genes between cucumber and other species were also detected and visualized by JCVI v 0.8.4 on the basis of the species phylogenetic tree, which was initially constructed with 77 single copy genes from 23 species by using OrthoFinder v 2.5.4. The divergence time in each node was calibrated by r8s v 1.7.0 with the divergence time of *A. trichopoda* and *V. vinifera* at around 188 million years ago [23,24]. *A. trichopoda* and *V. vinifera* were set as the outgroup. The gene clusters were verified with plantiSMASH v 1.0 by using the default parameters [25].

### 3.3. Transient Transfection of Bi Gene of A. sinensis in N. benthamiana Leaves

The open reading frame (ORF) of *As03G2784* was amplified and then inserted into the binary vector of *p*Cambia1300-35S, resulting in a plasmid named *p*Cambia1300-35S-*As03G2784*. A positive colony was inoculated in LB media supplemented with kanamycin (50 ng/µL) and rifampicin (30 ng/µL), and then cultured at 28 °C overnight after the plasmid *p*Cambia1300-35S-*As03G2784* was transformed into the strain EHA105 of *Agrobacterium tumefaciens*. The culture was incubated at 28 °C for 3 h until the OD600 of the culture reached 0.5, using induction media containing MgCl_2_ (10 mmol/L), MES (10 mmol/L), and acetosyringone (150 mmol/L) [26]. 

Subsequently, the culture was injected into 5-week-old *N. benthamiana* leaves. The *Agrobacterium*-transformed leaves of *N. benthamiana* were harvested after 5 days. Nine leaves of *N. benthamiana* from the control and treatments were harvested, with fresh weights ranging from 7.12 g to 7.45 g. These leaves were crushed into fine powder in liquid nitrogen. The powder was extracted with 30 mL of methanol for 5 min under vigorous vortexing and was then subjected to 30 min of ultrasonic extraction. This extraction procedure was repeated three times. Finally, the subsequent extraction was carried out in a chamber with analytical-grade methanol. 

Chlorophyll was removed using a cartridge of solid phase extract and the re-suspension liquid of the metabolites from the extraction was analyzed by using gas chromatography–mass spectrometry (GC-MS, Agilent 7820A/5977E, Santa Clara, CA, USA). Separation of the samples by gas chromatography was carried out using an HP-5MS 5% phenyl methyl siloxane column (30 m × 0.25 mm × 0.25 µm) (Phenomenex, Torrence, CA, USA). The parameters were set as the following: injection volume, 1.0 µL; the front inlet temperature, 250 °C; no split ratio; the flow rate of the helium (carrier gas), 1.0 mL/min; the interface temperature, 280 °C; ionization of the compounds, electron impact (EI); emission current, 70 eV; the ion source temperature, 230 °C. The oven program commenced at 50 °C and increased to 310 °C at a rate of 5 °C/min, then held for 10 min. The spectra were obtained over the mass range of *m*/*z* 30 to 550 and the relative contents of the compounds were determined by normalization [27]. The plasmid containing no coding sequence (the empty vector) was also similarly transfected into leaves of *N. benthamiana* and served as one control check. Further, the extract from healthy leaves of *N. benthamiana* was also set as another control check. Due to the high similarity of the MS chromatograms (EIC) between these two control checks, only the EIC of the treatment with the empty vector is shown in the main text (Figure 4c) and the EIC of the extract from heathy *N. benthamiana leaves* is presented in the Supplementary Materials (Figure S1). Each treatment contained three biological replicates.

## 4. Conclusions

In summary, this study provided valuable insights into the evolution and distribution of bitter (*Bi*) genes and their clusters in cucurbitacin-producing plants. The identification of 19 *Bi* genes in various species and a macroevolutionary tree of 23 species indicated that *Aquilaria sinensis* is the earliest origin of plant *Bi* gene clusters among them, which sheds light on the genetic basis of cucurbitacin biosynthesis. Additionally, the functional validation of the *Bi* gene in *A. sinensis* further supports its role in promoting cucurbitadienol biosynthesis. The distinct arrangements of *Bi* gene clusters in Cucurbitaceae, Begoniaceae, and *A. sinensis* suggest complex evolutionary dynamics in plant *Bi* gene cluster formation. These findings shed light on the genetic basis and evolutionary dynamics of plant *Bi* gene clusters and contribute to understanding cucurbitacin production, even offering genetic resources for potential enhancements in various plant species.

## Data Availability

The data presented in this study are available upon request from the corresponding author.

## Supplementary Materials

The following supporting information can be downloaded at: https://www.mdpi.com/article/10.3390/plants13020260/s1, Table S1: The protein sequences of *Bi* genes used in this study; Figure S1: The raw images of MS chromatograms (EIC) of *N. benthamiana* transiently transfected with *As03G2784*; Figure S2: The raw images of MS chromatograms (EIC) of *N. benthamiana* transiently transfected with *As03G2784* in Figure 4a and Figure S1a; Figure S3: The raw images of MS chromatograms (EIC) of the cucurbitadienol standard in Figure 4b and Figure S1b; Figure S4: MS chromatogram of *N. benthamiana* transiently transfected with empty vector only in Figure 4c and Figure S1c; Figure S5: MS chromatogram of the extract from the healthy leaves of *N. benthamiana* in Figure S1d.

## References

[1] Chen J., Chiu M., Nie R., Cordell G., Qiu S. (2005). Cucurbitacins and cucurbitane glycosides: Structures and biological activities. Nat. Prod. Rep..

[2] Balkema-Boomstra A.G., Zijlstra S., Verstappen F.W.A., Inggamer H., Mercke P.E., Jongsma M.A., Bouwmeester H.J. (2023). Role of cucurbitacin C in resistance to spider mite (*Tetranychus urticae*) in cucumber (*Cucumis sativus* L.). J. Chem. Ecol..

[3] Kong C., Liang W., Yang X., Zhang M., Hu F. (2004). Mechanism of *Aulacophora femoralis* chinensis Weise feeding behavior and chemical response of host *Cucumis sativus* L.. Chin. Sci. Bull..

[4] Zou C., Liu G., Liu S., Liu S., Song Q., Wang J., Feng Q., Su Y., Li S. (2018). Cucurbitacin B acts a potential insect growth regulator by antagonizing 20-hydroxyecdysone activity. Pest Manag. Sci..

[5] Mukherjee P.K., Singha S., Kar A., Chanda J., Banerjee S., Dasgupta B., Haldar P., Sharma N. (2022). Therapeutic importance of Cucurbitaceae: A medicinally important family. J. Ethnopharmacol..

[6] Huang S., Li R., Zhang Z., Li L.I., Gu X., Fan W., Lucas W.J., Wang X., Xie B., Ni P. (2009). The genome of the cucumber, *Cucumis sativus* L.. Nat. Genet..

[7] Shang Y., Ma Y., Zhou Y., Zhang H., Duan L., Chen H., Zeng J., Zhou Q., Wang S., Gu W. (2014). Biosynthesis, regulation, and domestication of bitterness in cucumber. Science.

[8] Zhou Y., Ma Y., Zeng J., Duan L., Xue X., Wang H., Lin T., Liu Z., Zeng K., Zhong Y. (2016). Convergence and divergence of bitterness biosynthesis and regulation in Cucurbitaceae. Nat. Plants.

[9] Mei W., Lin F., Zuo W., Wang H., Dai H. (2012). Cucurbitacins from fruits of *Aquilaria sinensis*. Chin. J. Nat. Med..

[10] Chen C.H., Kuo T.C.Y., Yang M.H., Chien T.Y., Chu M.J., Huang L.C., Lo H.F., Jeng S.T., Chen L.F.O. (2014). Identification of cucurbitacins and assembly of a draft genome for *Aquilaria agallocha*. BMC Genom..

[11] Ding X., Mei W., Lin Q., Wang H., Wang J., Peng S., Li H., Zhu J., Li W., Wang P. (2020). Genome sequence of the agarwood tree *Aquilaria sinensis* (Lour.) Spreng: The first chromosome-level draft genome in the Thymelaeceae family. GigaScience.

[12] Yu T., Cui H., Li J., Luo Y., Jiang G., Zhao H. (2023). Enzyme function prediction using contrastive learning. Science.

[13] Mahram A., Herbordt M.C. (2015). NCBI BLASTP on high-performance reconfigurable computing systems. ACM T. Reconfig. Technol..

[14] Finn R.D., Clements J., Eddy S.R. (2011). HMMER web server: Interactive sequence similarity searching. Nucleic Acids Res..

[15] Mistry J., Chuguransky S., Williams L., Qureshi M., Salazar G.A., Sonnhammer E.L.L., Tosatto S.C.E., Paladin L., Raj S., Richardson L.J. (2021). Pfam: The protein families database in 2021. Nucleic Acids Res..

[16] Li L., Chen X., Fang D., Dong S., Guo X., Li N., Campos-Dominguez L., Wang W., Liu Y., Lang X. (2022). Genomes shed light on the evolution of *Begonia*, a mega-diverse genus. N. Phytol..

[17] Zhang H., Ding X., Wang H., Chen H., Dong W., Zhu J., Wang J., Peng S., Dai H., Mei W. (2023). Systematic evolution of *bZIP* transcription factors in Malvales and functional exploration of *AsbZIP14* and *AsbZIP41* in *Aquilaria sinensis*. Front. Plant Sci..

[18] Cui J., Yang Y., Luo S., Wang L., Huang R., Wen Q., Han X., Miao N., Cheng J., Liu Z. (2020). Whole-genome sequencing provides insights into the genetic diversity and domestication of bitter gourd (*Momordica* spp.). Hortic. Res..

[19] Ma Y., Li D., Zhong Y., Zhong Y., Wang X., Li L., Osbourn A., Lucas W.J., Huang S., Shang Y. (2023). Vacuolar MATE/DTX protein-mediated cucurbitacin C transport is co-regulated with bitterness biosynthesis in cucumber. N. Phytol..

[20] Goodstein D.M., Shu S., Howson R., Neupane R., Hayes R.D., Fazo J., Mitros T., Dirks W., Hellsten U., Putnam N. (2012). Phytozome: A comparative platform for green plant genomics. Nucleic Acids Res..

[21] Yu J., Wu S., Sun H., Wang X., Tang X., Guo S., Zhang Z., Huang S., Xu Y., Weng Y. (2023). CuGenDBv2: An updated database for cucurbit genomics. Nucleic Acids Res..

[22] Nguyen L.T., Schmidt H.A., Von Haeseler A., Minh B.Q. (2015). IQ-TREE: A fast and effective stochastic algorithm for estimating maximum-likelihood phylogenies. Mol. Biol. Evol..

[23] Yang Z., Mei W., Wang H., Zeng J., Dai H., Ding X. (2023). Comprehensive Analysis of *NAC* Transcription Factors Reveals Their Evolution in Malvales and Functional Characterization of *AsNAC019* and *AsNAC098* in *Aquilaria sinensis*. Int. J. Mol. Sci..

[24] Tang H., Bowers J.E., Wang X., Ming R., Alam M., Paterson A.H. (2008). Synteny and collinearity in plant genomes. Science.

[25] Kautsar S.A., Suarez Duran H.G., Blin K., Osbourn A., Medema M.H. (2017). plantiSMASH: Automated identification, annotation and expression analysis of plant biosynthetic gene clusters. Nucleic Acids Res..

[26] Wang X.H., Gao B.W., Nakashima Y., Mori T., Zhang Z.X., Kodama T., Lee Y.-E., Zhang Z.-K., Wong C.-P., Liu Q.-Q. (2020). Identification of a diarylpentanoid-producing polyketide synthase revealing an unusual biosynthetic pathway of 2-(2-phenylethyl) chromones in agarwood. Nat. Commun..

[27] Yu Z., Dong W., Wang Y., Li W., Guo Z., Mei W., Dai H. (2023). Identification of aroma-active components from cultivated agarwood ‘Qi-Nan’ based on GC-O-MS combined with aroma extract dilution analysis. Flavour Fragr. J..

