# Supplementary material for: Aquilaria sinensis: An Upstart Resource for Cucurbitacin Production Offers Insights into the Origin of Plant Bitter (Bi) Gene Clusters
Source: Plants (Basel). 2024 Jan 16;13(2):260. doi: 10.3390/plants13020260 (PMC10819951; doi:10.3390/plants13020260)
Supplement: Supplementary file 1 [file plants-13-00260-s001.zip › Figures S1-S5.pdf]

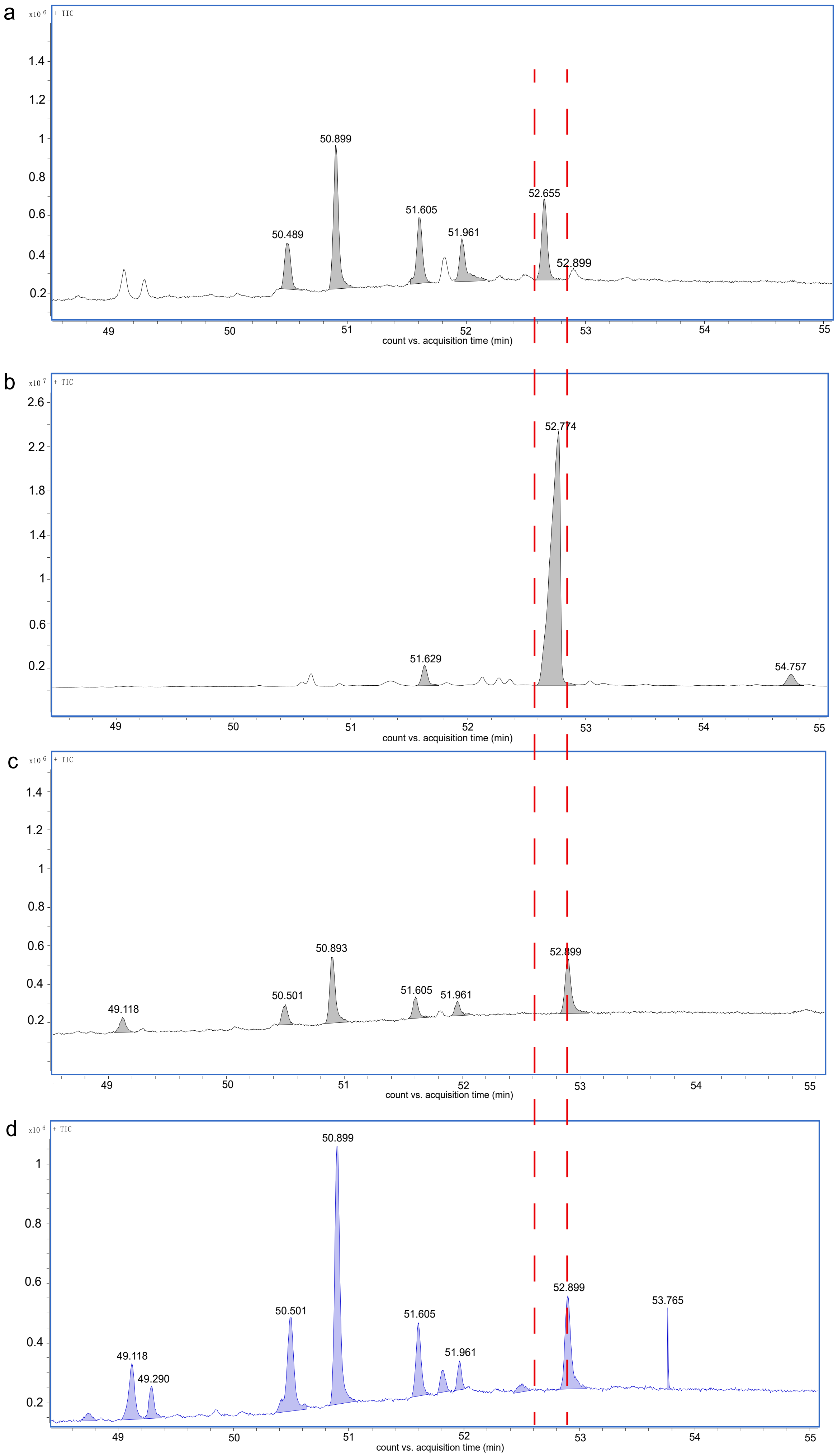

Figure S1. The raw images of MS chromatograms (EIC) of *N. benthamiana* transient transfected by *As03G2784*. (a) MS chromatograms (EIC) of *N. benthamiana* transiently expressing *As03G2784*. (b) MS chromatogram (EIC) of cucurbitadienol standard. (c) MS chromatogram of *N. benthamiana* transiently transfected with empty vector only.(d) MS chromatogram of extraction from healthy leaves of *N. benthamiana.*

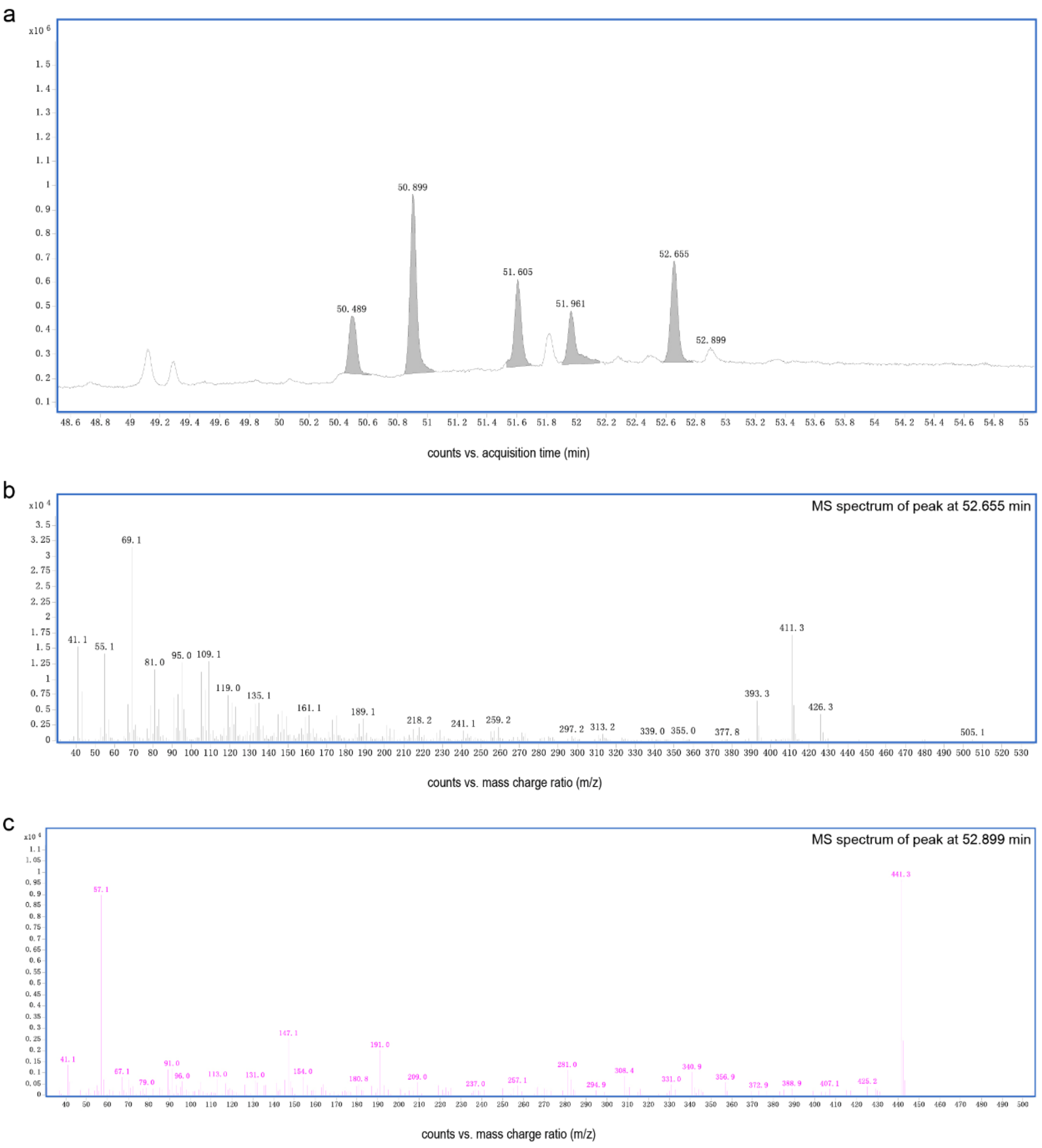

Figure S2. The raw images of MS chromatograms (EIC) of *N. benthamiana* transient transfected by *As03G2784* in Figure 4a and Figure S1a. (a) MS chromatograms (EIC) of *N. benthamiana* transiently expressing *As03G2784* (*Bi*). (b) ES−MS spectrum of peak at 52.655 min in Figure 4a and Figure S1a. (c) ES−MS spectrum of peak at 52.899 min in Figure 4a and Figure S1a.

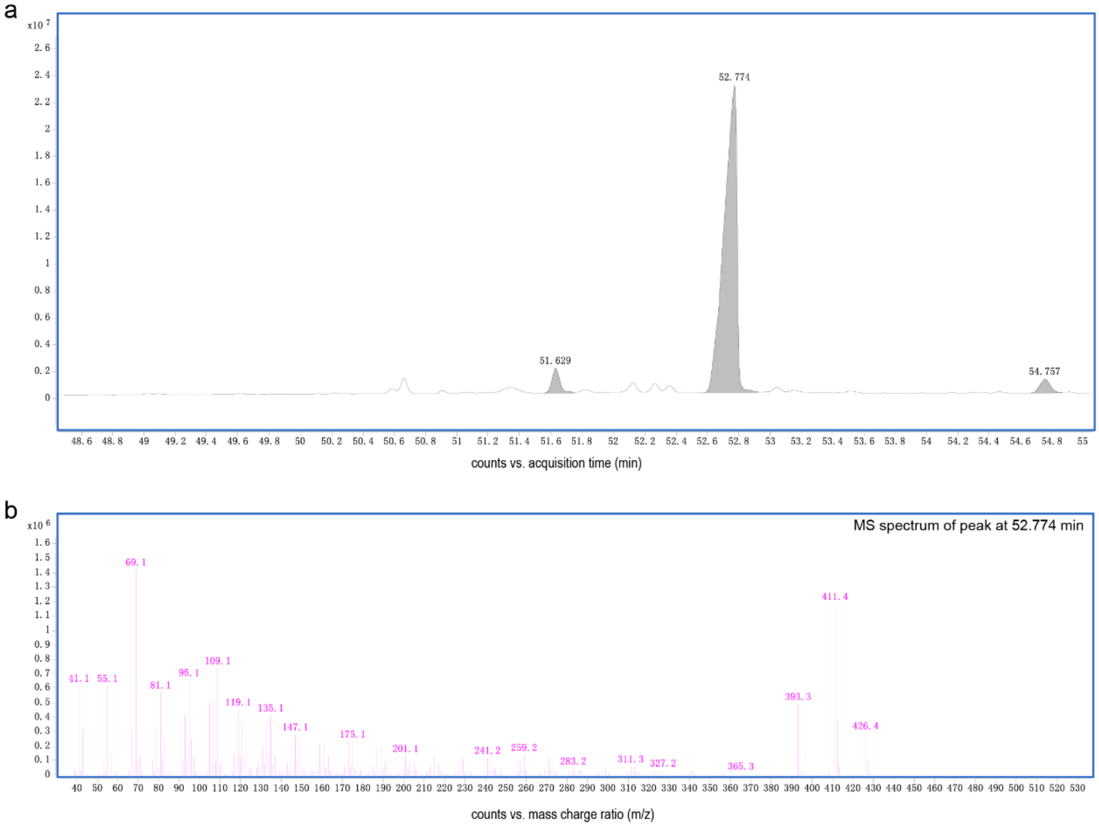

Figure S3. The raw images of MS chromatograms (EIC) of cucurbitadienol standard in Figure 4b and Figure S1b. (a) MS chromatograms (EIC) of cucurbitadienol standard in Figure 4b and Figure S1b. (b) ES−MS spectrum of peak at 52.774 min in Figure 4b and Figure S1b.

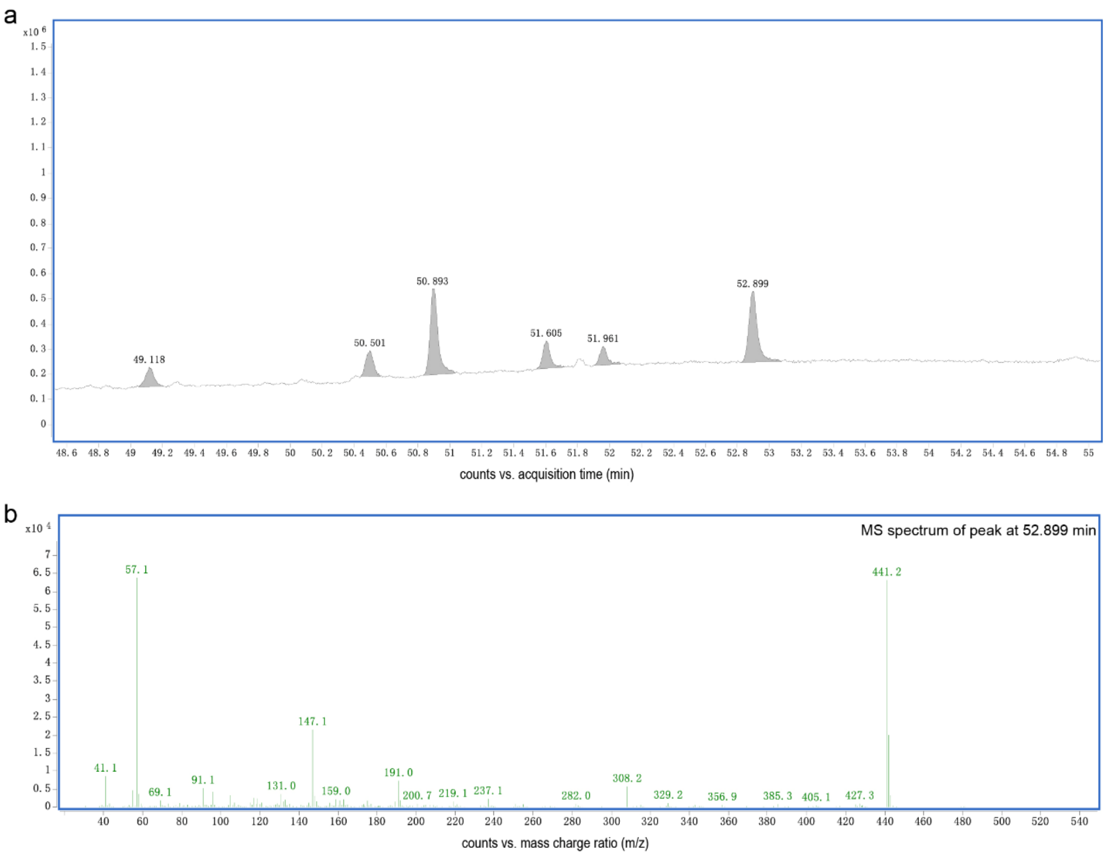

Figure S4. MS chromatogram of *N. benthamiana* transiently transfected with empty vector only in Figure 4c and Figure S1c. (a) MS chromatograms (EIC) of *N. benthamiana* transiently transfected with empty vector only in Figure 4c and Figure S1c. (b) ES−MS spectrum of peak at 52.899 min in Figure 4c and Figure S1c.

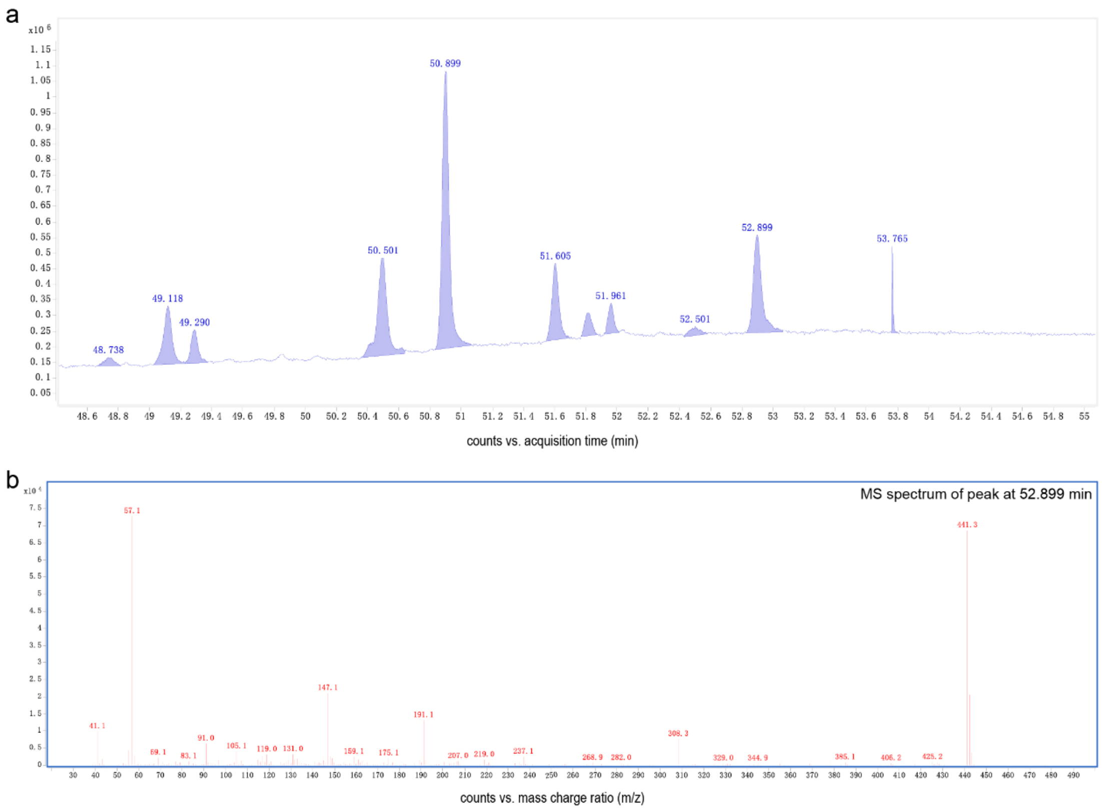

Figure S5. MS chromatogram of extraction from healthy leaves of *N. benthamiana* in Figure S1d. (a) MS chromatograms (EIC) of extraction from healthy leaves of *N. benthamiana*. (b) ES−MS spectrum of peak at 52.899 min in Figure S1d and Figure S5a.
